# Characterizing Residual Stresses in Additively Manufactured Alloys at the Cornell High Energy Synchrotron Source

**DOI:** 10.1063/4.0001002

**Published:** 2025-10-27

**Authors:** Kelly E Nygren, Christopher Budrow, Paul Shade, Peter Ko, Amlan Das, Diwakar Naragani, Arthur Woll, Matthew P Miller

**Affiliations:** 1 Cornell University, Ithaca, New York, USA; 2 Budrow Consulting LLC, Albany, New York, USA; 3 Air Force Research Laboratory, Dayton, Ohio, USA

## Abstract

One of the major challenges in metal additive manufacturing is the development of residual stresses due to the rapid heating and cooling cycles inherent in the process. These stresses can significantly impact the mechanical performance and structural integrity of additively manufactured components. To quantify these stresses, synchrotron X-ray diffraction provides a powerful, non-destructive technique for measuring internal lattice strains, both during fabrication and after heat treatments, to assess different processing pathways. High-energy synchrotrons offer the flux and penetrating power required to probe dense crystalline materials, such as engineering alloys. By mapping the elastic lattice strains within a sample, residual stresses can be calculated, providing critical data for validating complex thermal processing models. These models help accelerate the adoption of additively manufactured parts by enabling the optimization of print parameters and post-processing treatments. Residual stress measurements, particularly in additively manufactured metal components, have been a cornerstone of the Structural Materials Beamline (SMB) program at the Materials Solutions Network at the Cornell High Energy Synchrotron Source (MSN-C). As a defense research facility funded by the Air Force Research Laboratory, MSN-C focuses on transitioning synchrotron-based methods from academic research to practical engineering tools - a challenge endemic to the synchrotron community limiting industrial access to these measurements. The SMB beamline is equipped with both high-energy monochromatic (40–90 keV) and polychromatic (50–200 keV) X-ray capabilities, enabling the measurement of residual elastic lattice strains in component-sized parts using two primary techniques. The first, Angular X-ray Dispersive Diffraction (ADXRD), is a monochromatic technique that utilizes SMB’s recently acquired large-panel, direct X-ray area detector (Dectris Eiger16M CdTe), making it well-suited for thin-walled structures (less than 7 mm). The second, Energy X-ray Dispersive Diffraction (EDXRD), is a polychromatic technique that employs SMB’s 23-element energy dispersive detector system, allowing for deeper penetration—up to 40 mm in steel—making it ideal for larger component parts.

This talk will present the current state-of-the-art in residual stress measurements at SMB developed over the last 6 years, and the readiness of these methods for industrial applications. The complex microstructures of additively manufactured parts pose challenges across measurement techniques, leading to effective error bounds that will be discussed. Additionally, this presentation will cover the challenges associated with stress-relaxed lattice parameter determination in additively manufactured parts, particularly those arising from compositional and microstructural complexities, as well as strategies for improving measurement accuracy.

